# Supplementary material for: Large Thermal Motion in Halide Perovskites
Source: Sci Rep. 2017 Aug 24;7:9401. doi: 10.1038/s41598-017-09220-2 (PMC5571225; doi:10.1038/s41598-017-09220-2)
Supplement: Supplementary file 1 — Supplementary Document [file 41598_2017_9220_MOESM1_ESM.pdf]

Large Thermal Motion in Halide Perovskites (Supplementary Document)

T. A. Tyson[1,*], W. Gao[2], Y.-S. Chen[3], S. Ghose[4] and Y. Yan[5,*]

[1]Department of Physics, New Jersey Institute of Technology, Newark, NJ 07102
[2]Department of Chemistry, University of South Florida, Tampa, FL 33620
[3]ChemMatCARS, University of Chicago and Advanced Photon Source, Argonne National Laboratory, IL 60439
[4]National Synchrotron Light Source II, Brookhaven National Laboratory, Upton, NY 11973
[5]Department of Chemistry and Environmental Science, New Jersey Institute of Technology, Newark, NJ 07102

*Corresponding Authors: T. A Tyson, e-mail: tyson@njit.edu and
Y. Yan, e-mail: yong.yan@njit.edu

I. Experimental and Modeling Methods

To synthesize single crystal of $CH_3NH_3PbI_3$, 3 mmol PbI_2 and 3 mmol CH_3NH_3I were prepared in gamma-butyrolactone (3mL, targeting for 1mol/L solution). This mixture was heated up to 60 °C to completely dissolve any insoluble residue. The solutions were quickly filtered using a PTFE filter with 200 nm pore size. Two milliliters of the filtrate were placed in a vial which was then kept in an oil bath undisturbed at 110 °C for perovskites single crystal growth. All procedures were carried out under ambient conditions and a humidity of 55–57%. The crystals used for measurements were grown for 5 hrs. The obtained single crystals were dark with well- defined and highly reflective facets. For experiments involving powders, single crystals were ground into fine powders (below 500 mesh). All experiments were conducted on crystals from the same batch.

Specific heat measurements were conducted on warming from 300 K to 360 K and on cooling from 360 K to 170 K using the relaxation method in a Quantum Design PPMS system. Temperature steps of 0.2 K were utilized in the region of the transition near 329 K and each temperature point was measured three times and the average result is reported (Fig. 1). Approximately 10 minutes was required to measure each temperature data point. Hence the system was never in a quenched state.

Synchrotron single crystal x-ray diffraction measurements on ~15 μm diameter crystals were carried at the beamline 15-ID-B of the Advanced Photon Source (APS) at Argonne National Laboratory using a wavelength of 0.41328 Å. Refinement of the single crystal data was conducted using the program SHELXL [1] after the reflections were corrected for absorption (see Ref. 2). For the space group I4/mcm, we obtained significantly worse fitting agreement (R_1= 8.49 %, wR_2= 28.0 % and Goodness of Fit =1.26). Furthermore, for the I4/mcm space group, the C-N bond distance was found to be unstable without the use of constraints.

Pair distribution function experiments were conducted at beamline the XPD (28 ID) beamline at Brookhaven National Laboratory's National Synchrotron Light Source II using a wavelength λ = 0.18372 Å (67.486 keV). The data were measured using a Perkin Elmer detector with a sample to

detector distance of 204.08 mm. The range Q_{mim} = 0.23 Å^{-1} and Q_{max} = 25.1 Å^{-1} was used in data reduction. The methods utilized for analysis of the PDF data are described in detail in Refs. [1,3]. For the fits in R-space covered the range: 2.75 < r < 60 Å. The time interval between temperature points was ~15 minutes making the experimental conditions consistent with those of the heat capacity measurements.

A single crystal was used for the x-ray absorption measurements. XAFS spectra were collected at APS beamline 13-ID-E. Four Pb L3-Edge XAFS data sets (12785 to 13788 eV) were collected in fluorescence mode using a four Vortex detectors with data corrected for deadtime. The averaged data sets were used in the analysis. Reduction of the x-ray absorption fine-structure (XAFS) data was performed using standard procedures [4]. In the XAFS refinements, to treat the atomic distribution functions on equal footing, the Pb spectra were modeled in R-space by optimizing the integral of the product of the radial distribution functions and theoretical spectra with respect to the measured spectra. Specifically, the experimental spectrum is modeled by $\chi(k) = \int \chi_{th}(r,k) 4\,\pi\, r^2 g(r) \varrho\, dr,$ where $\chi_{th}(r,k)$ is the theoretical spectrum, ϱ is the bond number density. To search for asymmetry a split Gaussian distribution $4\,\pi, r^2 g(r)\varrho$ = n(r) is modeled by the function: $A\,\exp[-(r-r_0)^2/(2\sigma_1^2)]$ for r < r_0 and $A\,\exp[-(r-r_0)^2/(2\sigma_2^2)]$for r > r_0. The parameter r_0 is the peak position, and σ_1 and σ_2 are the left and right widths of the asymmetric Gaussian function. Theoretical spectra for atomic shells [5] were derived from the room temperature crystal structure. The k-range 2.79 < k < 11.5 Å^{-1} and the R-range 1.74 < R < 3.81 Å were in the structural refinement. A value of S_0^2=0.9 was used (accounting for electron loss to multiple excitation channels).

Molecular dynamics (MD) simulations were conducted utilizing the VASP code implementing projector-augmented wave (PAW) potentials [6] to properly account for the bonding and atomic motion (see also Ref. [7]). The LDA exchange functional (Ceperly and Alder as parameterized by Perdew and Zunger [8]) were used with a 400 eV energy cutoff. These potentials are all standard PAW optimized potentials within VASP. A 2x2x1 orthorhombic supercell (based on the orthorhombic cell of Ref. [9]) with 192 atoms was utilized to full models the room temperature without imposing structural constraints.

For separate MD simulations, the system temperature was set at 200, 300, 400 and 500 K utilizing the (N V T) ensemble. Time steps of 0.5 fs were carried out (to properly account for the motion of atoms within the $CH_3NH_3^+$ ions). No constraints on the atomic positions were utilized. The real atomic masses (no duteration of H atoms) were used in the simulations. An equilibration run of ~3300 steps (1.5 ps) was followed by as a second set of ~3300 time-steps. The latter run was utilized in the calculating the MD-derived properties shown below. For the defect structure with an MA ion and I atom removed, the same procedure was carried out as for the standard 300 K simulations. Note that the molecular dynamics simulation are conducted assuming no specific space group (P1 space group) and imposing no specific constraints on the atomic positions.

II. Results

Table S1. Structural Parameters from Single Crystal Refinement at Room Temperature

**Atoms**	**x**	**y**	**z**	Ueq ($Å^2$)×10^3
Pb	0	0	0.22462(2)	39.8(8)
I1	0	0	0.4718(13)	121 (3)
I2	0.2780(6)	0.2220(6)	0.2207(17)	112 (3)
N	0.4168(42)	0.0833(42)	0.4520(35)	26 (11)
C	1/2	0	0.3677(78)	76 (24)

N site occupancy = 50%

U_{ij} (Pb) 0.0390(9) 0.0413(10) 0.0000 0.0000 *

U_{ij} I1) 0.1666(44) 0.0298(25) 0.0000 0.0000 *

U_{ij} (I2) 0.0929(25) 0.1506(64) -0.0548(28) -0.0381(60) *

Space Group: I4cm (Z=4)
a = 8.9406 (13) Å, c = 12.6546 (25) Å, Dx = 4.071 g/cm^3
Measurement Temperature: 296 K
Crystal Dimensions: ~15 μm (diameter)
Wavelength: 0.41328 Å,
BASF twin parameter: 0.47(12)
Absorption Coefficient: 13.92 mm^{-1}
EXTI extinction parameter:0.0225(72)
F(000) = 1040
Reflections Collected: 14538
2θmax: 34.2°
$-12 \leq h < 11$, $-11 \leq k < 12$, and $-16 \leq l < 18$,
Number of Unique Observed Reflections $F_o > 4\sigma(F_o)$: 457
Number of fitting parameters: 20
Amplitude of Max Peak in Final Difference map: 2.63 e-/ $Å^3$ (N)
R_1 = 7.14 %, wR_2 = 19.0%, Goodness of Fit = 1.09

$$R_1 = \sum ||F_0| - |F_c|| / \sum |F_0|$$

$$wR_2 = \sum w(F_o^2 - F_c^2)^2) / \sum w(F_o^2)^2$$

* Atomic displacement parameters U_{ij} ($Å^2$) are in the order: U_{11}, U_{33}, U_{12}, and U_{23}.

Table S2. Bond Distances

**Bond Type**	**Bond Distance** (Å)
Pb-I1	3.127 (16)
	3.200 (16)
Pb-I2	3.181 (1)
<Pb-I>	3.169
C-N	1.5 (1)

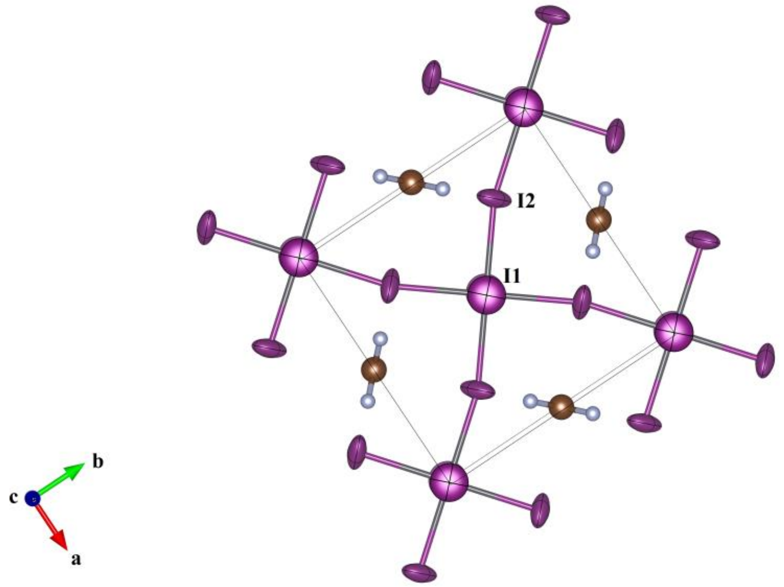

Fig. S1. View of the structure with the a-b plane in the plane of the figure.

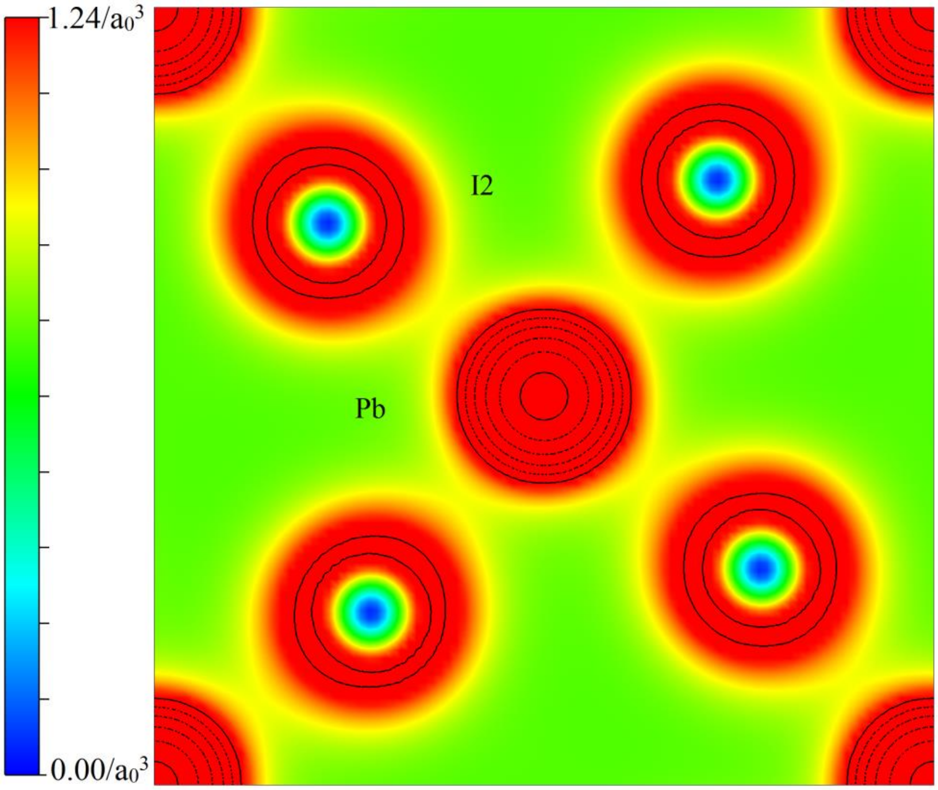

Fig. S2. Charged density (electrons/a_0^3) at z = 0.2246 based on DFT simulations using the experimental lattice parameters and atomic positions (Table S1). Note the weak charge build up between the Pb and I2 sites and the more extended distribution of the charge about the I sites compared to the Pb sites. This is distinctly different from the more localized behavior of charge on the O atoms in complex oxides (see for example Fig. 8 in Ref. [10]).

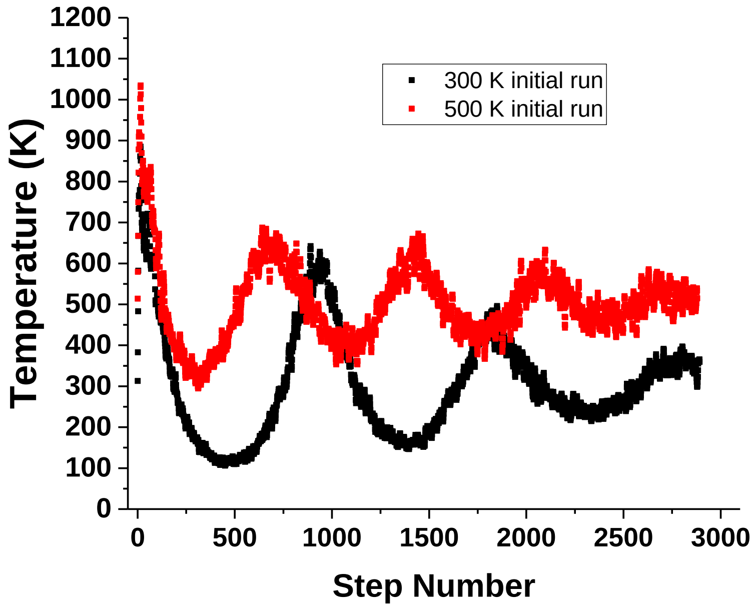

(a)

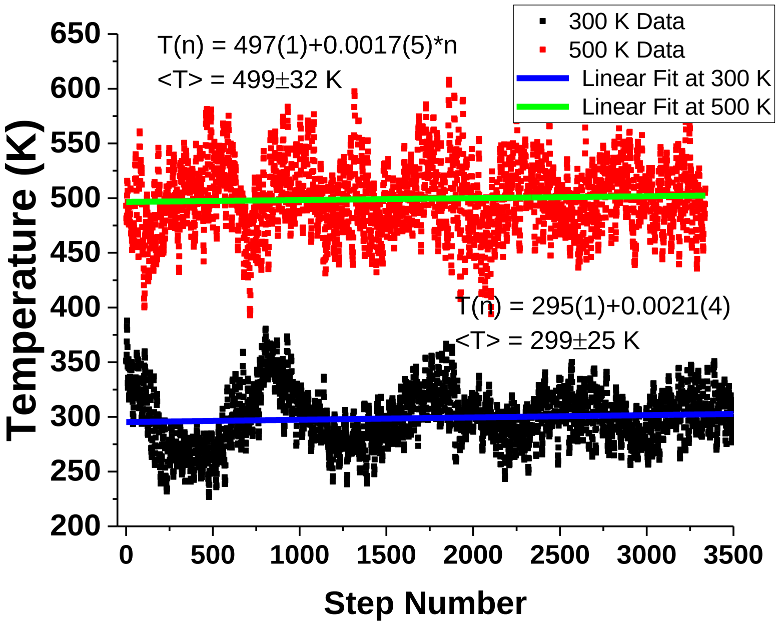

(b)

Fig. S3. Representative molecular dynamics simulation at 300 K and 500 K. Panel (a) gives the results from the first set of ~3000 time-steps (each step is 0.5 fs) each for the 300 K and 500 K runs. The results from the second set of ~3500 time-steps are given in (b). Thes results from these latter runs were used to compute all properties given in this work. Fits to the data as a straight line are also given to probe the level of the temperature convergence.

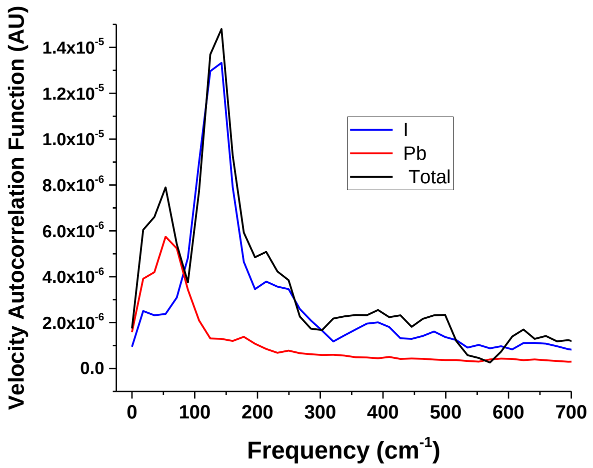

Fig. S4. Velocity autocorrelation function derived from the molecular dynamics simulation giving the phonon DOS for the I and Pb sites. Note the low-frequency positions for the Pb (~40 cm^{-1}) and I (~130 cm^{-1}) related modes in this systems. This is consistent with a mechanically soft material as found experimentally.

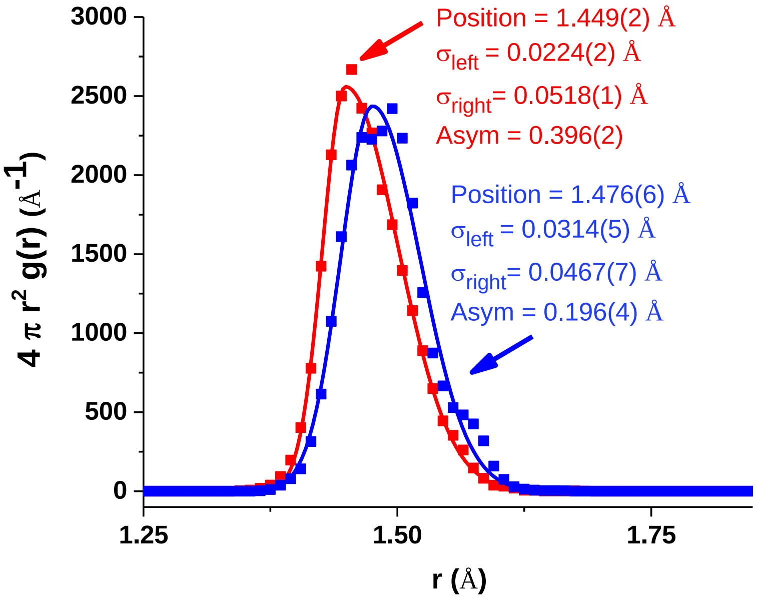

Fig. S5. Radial distribution function for the C-N pair of methylamine hosted in the $CH_3NH_3PbI_3$ system (red line) and in an isolated cell (same supercell without Pb and I ions and the 15,002 time-steps). Note the reduction of the asymmetry and expansion of the C-N bond length in the isolated methylamine molecule. Fits to the MD simulation with a left (σ_{left}) and right (σ_{right}) sided Gaussian (continuous at the peak) were used to assess the asymmetry (Asym = $\frac{|\sigma_{left}-\sigma_{right}|}{\sigma_{left}+\sigma_{right}}$).

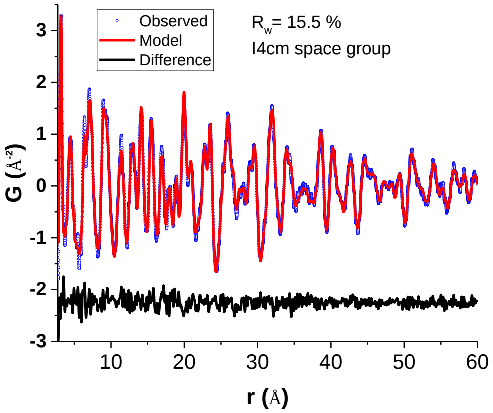

(a)

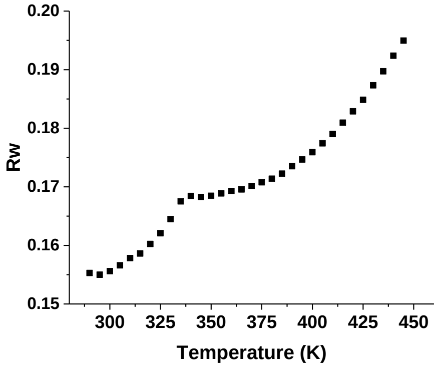

(b)

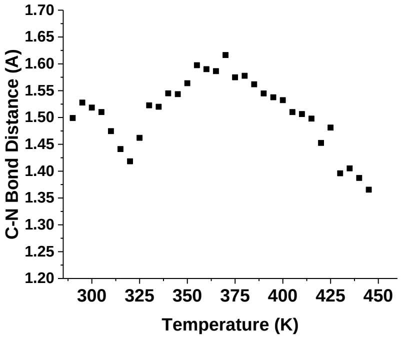

(c)

Fig. S6. The model derived from the room temperature single crystal structure (Table S1) was found to fit the PDF data up to 450 K. All data were fit over the range 2.75 to 60 Å to the I4cm space group model. A typical fit of the data taken at 300 K is shown in (a). R_w values for the fits between 280 and 450 K are shown in (b). The extracted bond C-N bond distance is given in (c) serve to show the goodness of the fit, data quality and data calibration over the entire temperature range. The values should be compared to the results of in Fig. S3 and the results in Table S2.

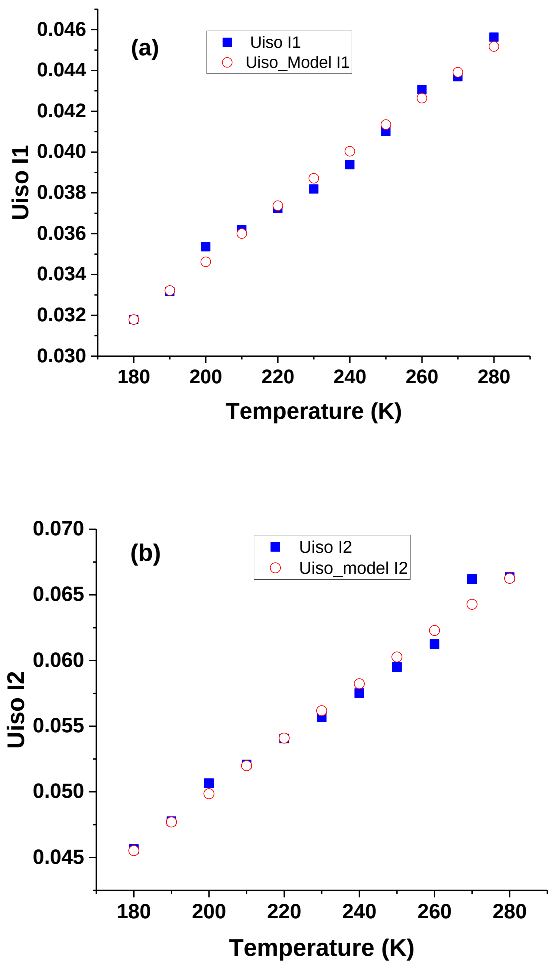

Fig. S7. Fits for the iodine U_{iso} (Å^2) parameters (below T*) for I1 (a) and I2 (b) to the PDF data using a single particle potentials V(u) assuming a Boltzmann energy distribution. The potential form used was $V_0 + \alpha/2\ u^2 + \gamma\ u^4$ assuming a spherical shape of the potential. The extracted one particle potentials are given in Fig. 4(c) of the text. Closed symbols correspond to the U values from the scattering data, and open symbols are from the potential fits.

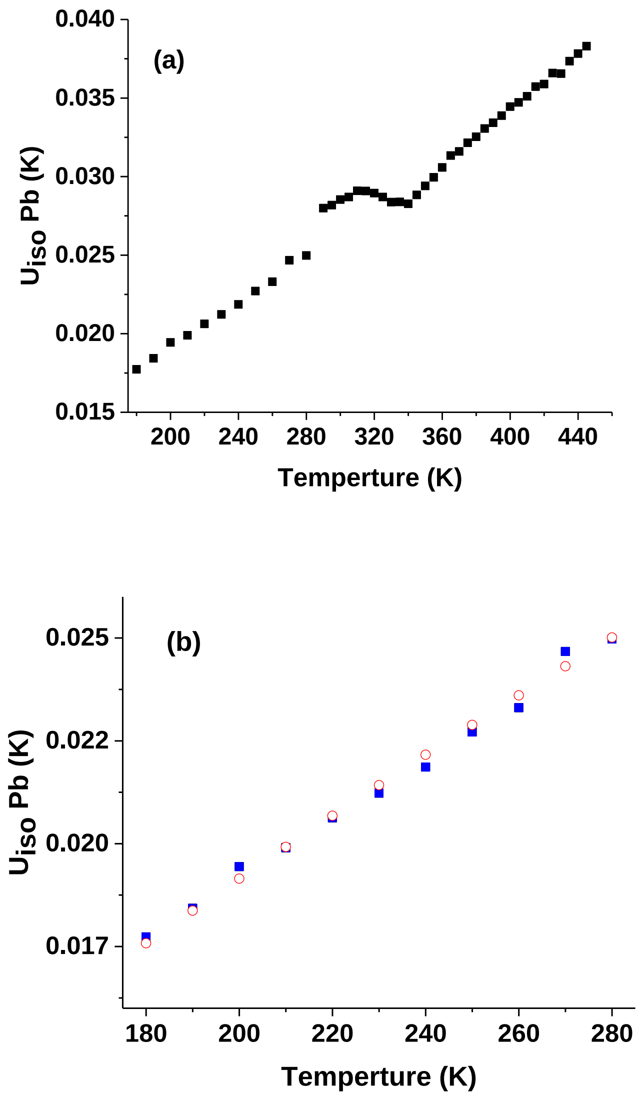

Fig. S8. Pb U_{iso} (Å^2) parameters and fits below T*. (a) Thermal parameters for the Pb site showing a bump in a broad region from ~280 K to ~340 K. (b) Potential fit for Pb using the same form as that for the I sites. The symbols have the same meaning as in Fig. S7.

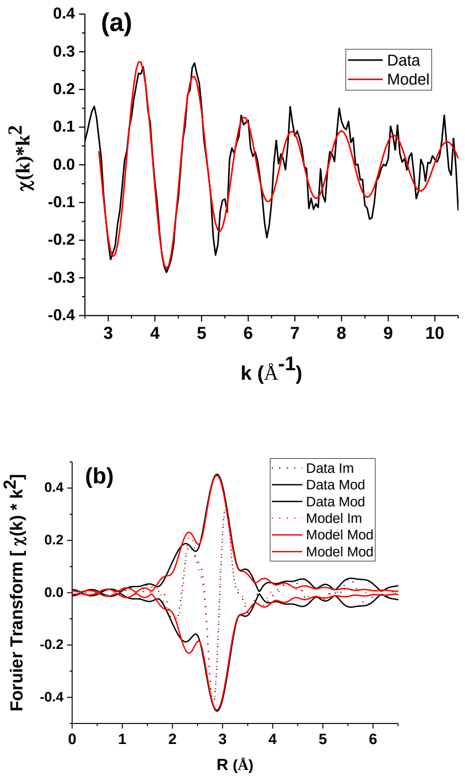

Fig. S9. Fits for the Pb L3 XAFS data shown in k-space (panel (a)) and in R-space (panel (b)). The fit shown is for a Pb-I bond distribution represented by an asymmetric Gaussian function as in Fig. 3(b) and Fig. S5.

References

[1] (a) T. Yu, T. A. Tyson, P. Gao, T. Wu, X. Hong, S. Ghose, and Y.-S. Chen, Phys. Rev. B. **90**, 174106 (2014).

(b) P. Muller, R. Herbst-Irmer, A. L. Spec, T. R. Schneider, M. R. Schneider, Crystal Structure Refinement: A Crystallographer's Guide to SHELXL; Oxford Press: Oxford, 2006. (b) Sheldrick, G. M. SHELX-76: Program for Crystal Structure Determination; Cambridge University: Cambridge, 1976.

[2] SADABS: Area-Detector Absorption Correction; Siemens Industrial Automation, Inc.: Madison, WI, 1996.

[3] (a) R. B. Neder and Th. Proffen, Diffuse Scattering and Defect Structure Simulations, (Oxford University, Oxford, 2008).

(b) T. Egami and S. L. J. Billinge, Underneath the Bragg Peaks: Structural Analysis of Complex Materials, (Pergamon, Amsterdam, 2003).

(c) Th. Proffen, S. J. L. Billinge, T. Egami and D. Louca, Z. Kristallogr **218**, 132 (2003).

(d) V. Petkov, in Characterization of Materials, (John Wiley and Sons, Hoboken, 2012).

[4] (a) T. A. Tyson, M. Deleon, S. Yoong, and S. W. Cheong, Phys. Rev. B: Condensed Matter and Materials Physics **75**, 174413 (2007).

(b) B. Ravel and M. Newville, J. Synchrotron Rad. **12**, 537 (2005); *X-Ray Absorption: Principles, Applications, Techniques of EXAFS, SEXAFS and XANES*, edited by D. C. Konningsberger and R. Prins (Wiley, New York, 1988).

[5] A. L. Ankudinov and J. J. Rehr, Phys. Rev. B **56**, R1712 (1997).

[6] (a) P. E. Blöchl, Phys. Rev. B **50**, 17953 (1994).

(b) G. Kresse, and J. Joubert, Phys. Rev. B **59**, 1758 (1999).

[7] T. A. Tyson, T. Wu, H. Y. Chen, J. Bai, K. H. Ahn, K. I. Pandya, S. B. Kim and S. W. Cheong, J. Appl. Phys. **110** , 084116 (2011).

[8] J. P. Perdew and A. Zunger Rev. B **23**, 5048 (1981).

[9] M. T. Weller, O. J. Weber, P. F. Henry, A. M. Di Pumpo and T. C. Hansen, Chem. Comm. **51**. 4180 (2015).

[10] T. A. Tyson, T. Wu, K. H. Ahn, S.-B. Kim and S.-W Cheong, Phys. Rev. B: Condens. Matter Mater. Phys. **81**, 054101 (2010).
